# Exploring the underutilized novel foods and starches for formulation of low glycemic therapeutic foods: a review

**DOI:** 10.3389/fnut.2023.1162462

**Published:** 2023-04-21

**Authors:** Vijayalakshmi Dega, Mrunal Deepak Barbhai

**Affiliations:** Department of Food Science and Nutrition, University of Agricultural Sciences, Bengaluru, Karnataka, India

**Keywords:** low glycemic index, nutraceutical starch, processing by-products, underutilized, therapeutic

## Abstract

Rising incidences of life-style disorders like obesity, diabetes and cardiovascular diseases are a matter of concern coupled with escalated consumption of highly refined and high energy foods with low nutrient density. Food choices of consumers have witnessed significant changes globally with rising preference to highly processed palatable foods. Thus, it calls food scientists, researchers and nutritionists’ attention towards developing and promoting pleasant-tasting yet healthy foods with added nutritional benefits. This review highlights selected underutilized and novel ingredients from different food sources and their by-products that are gaining popularity because of their nutrient density, that can be employed to improve the nutritional quality of conventionally available empty-calorie foods. It also emphasizes on the therapeutic benefits of foods developed from these understudied grains, nuts, processing by-products of grains, fruits- and vegetable-byproducts and nutraceutical starches. This review aims to draw attention of food scientists and industrialists towards popularizing the utilization of these unconventional, yet nutrient rich foods sources in improving the nutritional profile of the conventional foods lacking in nutrient density.

## 1. Introduction

Recent decades across the globe and all the age groups, have shown marked increase in consumption of ultra-processed, processed, ready to eat convenience fried snacks, chips, and refined foods like desserts, bakery and confectionery. These foods lack nutrient density and are rich in calories, attributed to their high fat and/or sugar content, and salt (at certain instances). Thus, their excessive consumption combined with the sedentary lifestyle elevates risk of non-communicable diseases (NCDs) such as diabetes, obesity, cardiovascular disorders and so on (1). It was recorded in World Obesity Atlas (2) that by 2030 one in five women and one in seven men will have obesity (2). According to world health organization (WHO), globally there are 650 million adults, 340 million adolescent and 39 million children who are obese and it was estimated that 167 million people (including adults and children) will become overweight or obese by 2025 (3). WHO also indicated that kidney disease and diabetes caused around 2 million deaths in 2019 (4), globally there are 537 million adults suffering with diabetes, which is predicted to rise by 643 million by 2030 (5). Along with the higher intake of the calorie-rich foods, current diets and consumption patterns also lack dietary fiber, this can consequently contribute in escalating the prevalence of above said disorders, along with constipation and irritable bowel syndrome (6, 7).

Present situation with hiked incidences of these lifestyle disorders is creating health concerns amongst the consumers. Changes in dietary pattern and lifestyle modifications are major management keys to prevent and manage these NCDs. One such modification in dietary practices that can assist in prevention and management of NCDs include consumption of low glycemic index (GI), high fiber foods rich in bioactive compounds (8–10). Thus, buyers are now looking for, and choosing healthy, yet tasty snacks, indicating that their choices are shifting towards picking nutritious and palatable alternatives. This drives the food researchers, scientists and technologists in developing therapeutic, functional foods and low glycemic index foods (to reduce the calorie density) (11, 12). This growing interest in formulation of functional and healthy foods has created a recent research trend of applying novel food sources and processing by-products for formulation of therapeutic foods. These novel ingredients include, underutilized starches, by-products of grains, fruits and vegetables (such as brans, peel, skin, seeds, fiber) that have been reported as a concentrated source of nutrients having multiple health benefits. For instance, whole grain and dietary fiber (including resistance starch) consumption are linked to prevention in lifestyle associated disorders (6). Bran, including cereal bran (i.e., rice, wheat, oat), millet bran of both major (jowar, pearl-, finger-millet bran) and minor millet (barnyard-, foxtail-, little-, kodo-, proso-millet bran) are also reported to be a rich source of fiber, antioxidants, phytochemicals and minerals by several investigations documenting their nutritional richness (13–19). Vegetable and fruit wastes also have ample quantity of fiber, vitamins, minerals and phytochemicals thereby exhibiting health-promoting abilities (20–24).

Overall, these wastes or by-products are generated on a large-scale during food processing and their waste management is the major challenge (25). Despite the abundance of nutrients, usually, majority of these by-products are used as animal feed (26–28), however given their therapeutic benefits, they can also be used as functional ingredient in human food (29, 30). With this understanding, the current review highlights some of these selected underutilized sources based on their nutritional profile with special emphasis on applying these ingredients for formulation of low glycemic index (GI) foods. The review also tries to draw attention on sensory and therapeutic properties of the novel low GI foods.

## 2. Potential novel food sources and their nutritional profile

Underutilized cereals and grains, nuts and oilseeds, fruits and vegetables possess good nutritional profile and thereby find application in development of the low GI therapeutic foods. Apart from these underutilized foods, even their processing by-products like bran, vegetable stems, fruit pomace, skin, peel, seeds and so on have can be employed as functional ingredient in development of value added, nutrient dense, therapeutic foods (14, 20–27, 29, 31, 32). This section discusses the nutritional profile of some of selected potential underutilized ingredients and their by-products that can find application in development of low GI foods. Table 1 summarizes nutritional profile of these selected novel ingredients.

**Table 1:** Nutritional profile of selected underutilized novel food sources.

Novel food sources	Nutrients									References
	Moisture (%)	Protein (%)	Fat (%)	Ash (%)	TDF (%)	CHO (%)	Iron (mg/100g)	Calcium (mg/100g)	Zinc (mg/100g)	
Pseudocereals										
Amaranth	3.62–12.41	13.27–21.50	5.60–10.90	2.65–3.2	2.7–20.6	59.2–76.50	7.61–9.33	159–181	1.6–2.87	Chandra et al. (33); Longvah et al. (34); Joshi et al. (35); Bekkering and Tian (36); Miranda-Ramos and Haros (37); Rodríguez et al. (38); Calderón de la Barca et al. (39)
Quinoa	10.43–12.62	13.11–17.51	5.50–7.5	2.6–2.82	7.0–26.5	53.65–69.0	4.51–7.51	32–198	1.8–3.31	Chandra et al. (33); Longvah et al. (34); Bekkering and Tian (36); Miranda-Ramos and Haros (37); Rodríguez et al. (38)
Chia	7.95	16.54–19.63	30.74-34.2	4.69	34.4–39.0	42.12	7.72	631	4.51	Bekkering and Tian (36); Miranda-Ramos and Haros (37)
Buckwheat	11.0	10.9–13.25	2.7–7.4	1.59	10–29.5	66–72.9	2.20–4.7	18–110.0	0.8–3.12	Chandra et al. (33); Joshi et al. (40); Bekkering and Tian (36); Rodríguez et al. (38); Pirzadah and Malik (41)
Minor millets										
Kodo millet	9.64	8.3	1.4	2.6–3.6	15–37.8	65–65.9	1.7–12	10–35	0.7	Hegde et al. (42); Devi et al. (43); Saha et al. (44); Chandra et al. (33); Das et al. (45); Srilekha et al. (46)
Proso millet	7.25	9.48–12.5	1.62–4.22	1.17–3.6	4.63–14.2	70.4–75.06	2.34–10	4.24–28	1.09–2.22	Devi et al. (43); Saha et al. (44); Chandra et al. (33); Habiyaremye et al. (47); Das et al. (45); Shankaramurthy and Somannavar (48); Bekkering and Tian (36); Torbica et al. (49)
Foxtail millet	9.59–9.93	8.92–12.94	2.55–4.3	3.0–3.3	6.39–19.11	60.9-71.58	2.34–6	10–35.63	1.65–3.51	Devi et al. (43); Singh and Hathan(50); Chandra et al. (33); Saha et al. (44); Habiyaremye et al. (47); Das et al. (45); Shankaramurthy & Somannavar (48); Rodríguez et al. (38)
Barnyard millet	8.7 –9.63	6.2–11.6	2.2–5.8	2.1–4.7	13–21.65	65.5	3.77–18.6	2.17–22.0	3.0	Chandra et al. (33); Sharma et al. (51); Saha et al. (44); Habiyaremye et al. (47); Kaur and Sharma (52); Gowda et al. (53)
Legumes										
Horse gram	9.28–11.55	17.9–25.3	0.5–2.06	3.24–3.8	7.88	57.24–60.9	8.76	269.0	2.71	Bhartiya et al. (54); Longvah et al. (34); Palai et al. (55)
Bambara beans	6.08–8.91	18.74–40.0	1.4–9.7	3.46–4.26	18.24–27.57	53.71–69	1.4–4.7	50.0	2.4–2.8	Gulzar and Minnaar (56); Oyeyinka et al. (57); Tan et al. (58); Hamadou et al. (59)
Cow pea	10.59–10.80	25.38–27.56	0.–79-7.57	3.42–6.84	14.03	45.64–59.29	0.20–0.24	9.0–36.0	0.7–.08	Gondwe et al. (60); Hamadou et al. (59)
Nuts and seeds										
Fox nut	11.2–34.7	7.2–11.40	0.3–0.63	0.58–15.21	0.80	57	0.12–0.8	0.18–9.5	0.015–0.9	National Institute of Food Technology Entrepreneurship and Management (61); Biswas et al. (62); Liaquat et al. (63)
Cereal and millet by-products										
Rice bran	4.30–5.2	6.4–20.90	4.7–20.85	4.92–14.19	21.0–49.19	36.63–77.13	18.54–28.10	52.10–57.0	6.02–6.04	Bagheri and Seyedein (64); Bhosale and Vijayalakshmi (15); Kaur et al. (65); Kalpanadevi et al. (66); Sapwarobol et al. (67); Ranjan et al. (27)
Wheat bran	7.2–12.7	5.69–25.59	2.95–4.62	2.12–8.10	33.40–63.0	26.8–75.0	1.9–34.0	73–78.3	8.3–14.0	Shenoy and Prakash (68); Butt et al. (69); Vitaglione et al. (70); Bisoi et al. (71); Onipe et al. (72); Kaur et al. (65); Dhillon and Tanwar (73); Duţă et al. (74); Sahin et al. (75)
Oat bran	7.69	5.54–18.0	1.00–10.59	1.45–2.89	14–25.2	33.0–66.22	3.5–5.41	47.0–58.0	3.11	Vitaglione et al. (70); Kaur et al. (65); Kristek et al. (76); Duţă et al. (74); Sahin et al. (75); Mustafa et al. (77)
Kodo bran	1.76–7.07	4.92–5.68	2.8–5.28	5.33–7.74	48.42–61.52	79.84–80.38	20.45	76.09	2.13	Bisoi et al. (71); Sarma et al. (78); Barbhai and Hymavathi (13)
Proso bran	1.88–7.32	1.78–26.33	1.92–8.96	5.77–8.44	34.74–70.53	69.64	60.74	37.87	5.59	Bisoi et al. (71); Mustač et al.(79); Barbhai and Hymavathi (13)
Foxtail bran	2.39–8.29	10.49–12.48	9.39–9.87	7.50–12.15	34.39	65.10	65.58	94.63	4.71	Liang et al. (80); Barbhai and Hymavathi (13)
Barnyard bran	2.41–6.79	5.15–7.70	4.04–7.85	9.11–10.02	37.26	72.03	8.87	62.26	3.83	Bisoi et al. (71); Barbhai and Hymavathi (13)
Underutilized fruits, vegetables and their by-products										
Water chestnut	9.68	2.82	0.28	2.38	5.65	81.71	1.76	35.05	2.96	Hussain et al. (81)
Apple seed	3–18.03	33.79–49.55	10.1–34	3.66–5.20	3.92–20.6	23.50–29.80	11.0–27.1	27–210	4.4	Yu et al. (82); Fromm et al. (83); El-Safy et al. (84); Dadwal et al. (85); Kumar et al. (20)
Mango seed/kernel	9.2-9.6	5.2–7.53	6.0–11.45	2.2–2.5	NA	36.2–69.77	11.9–12.4	25.2–450	1.10–5.60	Yatnatti et al.(24); Torres-León et al. (86); Masud et al. (87)
Prickly pear peel flour	6.58–9.11	3.3–3.69	2.12–5.17	10.81–14.57	33.0	49.6–73.41	117.5	929	100.5	Bouazizi et al. (88); Parafati et al. (89); El-Beltagi et al. (90)
*RDA for Indians (2020)*										
Men	–	54	–	–	40g/2,000 Kcal	130 g	19	1,000	17	ICMR-NIN (91)
Women	–	45.7	–	–			29		13.2	
Children (1–3 years)	–	11.7	–	–			8	500	3.0	
Children (4–9 years)	–	18.3–25.3	–	–			11–15	550–650	4.5–5.9	
Adolescents (10–18 years)	–	34.9–64.4	–	–			16–32	850–1,050	8.5–17.6	

TDF: total dietary fiber, CHO: carbohydrate, NA: not available.

### 2.1. Underutilized grains (cereals/pseudocereals, millets), legumes and their by-products

#### 2.1.1. Underutilized grains (cereals/pseudocereals, millets) and legumes

Pseudocereals, millets, and other underutilized grains need to be mainstreamed for providing dietary diversity and developing nutrient rich products apart from the major staple cereals (viz., rice, wheat and maize) and legumes (viz., pigeon peas, Bengal gram, green gram, chick peas, black matpe, kidney beans, peas, lentil) (92–94). Pseudocereals like amaranth (*Amaranth caudatus*) also called as ‘superfood’, quinoa (*Chenopodium quinoa* Willd) referred to as ‘Golden grains of Andes’, and millets as ‘nutri-cereals’ are good source of protein and essential amino acids (95, 96). Grain amaranth is known for its superior or equal protein and essential amino acid profile (viz., tryptophan, methionine, threonine, isoleucine, valine, phenylalanine) like staple cereals i.e., rice, wheat and maize (35, 97). Similarly, other pseudocereal namely buckwheat (*Fagopyrum esculentum* Moench.) has essential amino acids like lysine (5.9% of total protein), methionine (3.7% of total protein), tryptophan (1.4% of total protein) higher than rice (3.8%, 3.0%, 1.0% of total protein respectively), wheat (2.6%, 3.5%, 1.2% of total protein respectively), and maize (1.9%, 3.2%, 0.6% of total protein respectively) (40). Quinoa (17.51%) and chia (*Salvia hispanica* L.) seeds (19.63%) also contain higher protein than major staple cereals (37). Another nutritional benefit of consuming pseudocereals is absence of gluten, making it suitable for patients with celiac disease; and lower GI due to presence of total dietary fiber (TDF), resistance starch (RS) and SDS (slowly digestible starch) (98, 99). The TDF in quinoa was reported as 7–26.5%, while amaranth and buckwheat had 2.7–17.3 and 17.8 % respectively (35, 40); while Di Cairano et al. (98) revealed that the RS content of amaranth, buckwheat, and quinoa was 1.1g/100g, 0.5g/100g, 0.37g/100g, respectively. Thus, the pseudocereal like amaranth, chia seeds, buckwheat and quinoa, having ample TDF, RS and slow available glucose content, fall under low to moderate GI category. It was reported that expected glycemic index (eGI) of amaranth, chia seeds, buckwheat and quinoa ranged between low to moderate, with chia seeds having lowest eGI (28.53), followed by amaranth (47.65), buckwheat (52.35); while quinoa had moderate eGI (61.50). It was also reported that amaranth, chia, buckwheat and quinoa had RS content of 4.76, 0.08, 1.27, and 0.23%, respectively (100). In another study (98) the predicted GI for flours of amaranth, buckwheat and quinoa flours were indicated as 66.12, 50.74, 77.06 respectively. They also reported that the flours of amaranth, buckwheat and quinoa had total phenolic content (TPC) of ⁓ 1 mg gallic acid equivalents (GAE)/100 g, ⁓ 4.25 mg GAE/100g, ⁓ 1.70 mg GAE/100 g respectively; and TFC (total flavonoid content) of ⁓ 1.25 mg QE (quercetin equivalent)/100 g, ⁓ 3.25 mg QE/100 g, ⁓ 0.50 mg QE/100 g, respectively (98). Additionally, quinoa (184 μg/100 g) and amaranth (82 μg/100 g) have good folate content; while buckwheat (54 μg/100 g) and chia seeds (49 μg/100 g) had comparatively lower quantities of folate (36).

The protein content in minor millets namely foxtail (*Setaria italica*), kodo (*Paspalum scrobiculatum*), proso (*Panicum miliaceum*), and barnyard (*Echinochloa esculenta*) ranged between as 6–12.5% (33, 43–45, 47). In addition to protein, millets (major and minor) are also rich in dietary fiber, minerals and phenolic compounds, thus, they were termed as ‘*Shree Anna*’ during the presentation of Indian Budget, 2023 (101). Proso, kodo, foxtail and barnyard had TDF ranging from 8.5 to 37.8% with kodo millet having highest (37.8%) content and the mineral matter or ash content ranged between 1.9 and 4.4%. Minor millets have ample quantities of TPC ranging between 0.10 and 36.8 mg/100 g with kodo millet having highest quantities of phenols (36.8 mg/100 g) (33, 43–45, 47). Despite their nutrient density, millets are neglected grains, only major millets viz., sorghum (*Sorghum bicolor*), pearl millet (*Pennisetum glaucum*) and/or finger millet (*Eleusine coracana*) are consumed in many Indian states like Maharashtra, Karnataka, Orissa, Telangana and so on as staple grain after rice and wheat; however minor millets (kodo, proso, foxtail, barnyard, browntop) still remain greatly underutilized (13, 102–104).

Promising health benefits in preventing metabolic disorders upon consuming underutilized legumes like Bambara beans (*Vigna subterranea* (L.) Verdc.), Cowpea (*Vigna unguiculata* L. Walp.), horse gram [*Macrotyloma uniflorum Lam*. (Verdc.)], is attributed to their rich TDF, protein, and total phenolics, flavonoids and tannin profile (56, 59, 105). For Bambara beans and cowpea, the TDF, protein and fat ranged from 18.74–22.88 g/100 g, 14.03.27.57 g/100 g and 7.55–8.71 g/100 g, respectively. The authors described a strong positive correlation between frequent consumption of these underutilized grains and prevention of metabolic disorders like type II diabetes, obesity, high blood pressure and stroke (59). They also suggested that these legumes can assist in NCDs management and prevention due to the antioxidant activity of bioactive compounds viz., total phenolic content (TPC), total flavonoid content (TFC), and tannins apart from dietary fiber, that assist in lowering the oxidative stress associated with NCDs (59). Further, another study revealed that Bambara groundnut contained 61–69% CHO, 17–27% protein, 3.1–4.4% ash, 3.6–7.4% fat, and 3.3–6.4% fiber (106). In terms of horse gram, Prasad and Singh (105) reported that cotyledon had protein, fat, ash, TDF, CHO, soluble sugars, reducing sugars, viz., 22.6, 1.8, 2.9, 16.7, 66.9, 6.4 mg/100 g, and 538 mg/100 g, respectively. They also reported the TPC in horse gram cotyledon as 533.2 μg/g on dry weight basis, indicating the richness of bioactives present in horse gram.

Thus, reviving all these underutilized grains is essential for promoting health, wellbeing and developing low GI foods. The nutritional value of all these ingredients is summarized in Table 1.

#### 2.1.2. Grain processing by-products

Apart from grains, the by-products like bran, husk, bran rich fractions, obtained from cereal and millets during household or industrial processing are also remarkably rich in their nutritional and phytochemical profile. These agro-wastes are generally used as animal feed; however, given their nutritional and therapeutic benefits, their use is proposed as novel ingredient in development of functional human foods (16, 25, 29). The preventive role of whole grain consumption against NCDs are attributed to the dietary fiber and phytochemicals concentrated in the grains outer cover and bran (70). Cereal and millet bran have a generous amount of TDF, that can assist in reducing the GI of the foods. Besides the TDF content, they are also a concentrated source of minerals and phytochemicals that play a role in enhancing their hypoglycemic effect. Different cereal brans have varied nutritional profiles due to the varietal difference and other environmental factors. It was noted that wheat bran had TDF of 36.5–52.4 g/100 g, while oat bran, maize bran and rice bran had 18.1–25.2 g/100 g, 86.7 g/100 g, and 38.90 g/100 g, respectively (65, 70). Another study revealed that wheat, rice and oat bran had protein levels of 9.40, 11.83, and 15.08%, respectively (65). Patel (107) highlighted that cereal brans can be used in management of obesity, diabetes as proteins, oligosaccharides, RS, phenolic acids, flavonoids, lignans and other bioactive compounds are concentrated in cereal bran (107). Cereal bran also has beta-glucans (especially in oat and barley bran), phytosterols that exhibit hypocholesterolaemic and hypoglycemic effects (16). Wolevers et al. (108) reported that supplementation of instant oatmeal value added with β-glucan extracted from oat bran reduced the blood glucose levels postprandially.

Similarly, a comparison between four minor millet brans (viz., kodo, proso, foxtail and barnyard), indicated that kodo millet bran was rich in TDF (61.52%), phenols (449.27 mg GAE/100 g), flavonoids (22.37 μg) RE (Rutin equivalent)/g, phytic acid (630 mg/100 g); while minerals like iron and calcium were highest in foxtail millet bran (65.58 mg/100 g; 94.63 mg/100 g, respectively) and comparatively proso millet bran had higher zinc (5.59 mg/100 g) and potassium (630.83 mg/100 g) levels. The protein content (g/100 g) of bran viz., kodo, proso, foxtail and barnyard were 5.68, 13.04, 10.49, 7.70 g, respectively, with proso bran having highest protein content (13). Thereby the authors concluded that millets brans can be used as functional ingredient in food and pharma industry just like cereal brans (13). Liang et al. (80) reported that foxtail millet bran had good protein (12.48%), fat (9.39%), crude fiber (51.69%), and ash (7.50%) content; and the oil extracted from foxtail bran had oleic acid (13.0%), linolenic (66.5%), α-tocopherol (15.53 ± 0.31 mg/100 g oil) and γ-tocopherol (48.79 ± 0.46 mg/100 g oil). While kodo millet bran had 4.92% protein, 79.84% carbohydrate, 2.83% fat, 5.33% ash, and 48.42% TDF (78). The phenolic compounds are concentrated in brans and it was reported that foxtail bran had phenolic content (510.53 mg/100g) higher than foxtail whole grain (132.76 mg/100 g) (109). Bisoi et al. (71) indicated that fibers extracted from proso, barnyard, kodo, sorghum, finger millet bound glucose molecules and lowered the starch digestibility demonstrating hypoglycemic effect. Another study demonstrated that a diet fed with kodo millet bran to mice for 16 weeks prevented the increase in serum cholesterol, lipids and glucose, thereby improving glucose tolerance. Kodo bran supplementation also increased the beneficial gut bacteria viz., *Bifidobacteria*, *Lactobacillus* sp., *Roseburia* spp. and *A. muciniphila* (78). Altogether, millet brans are also condensed source of nutrients with equal or superior nutritional profile like major cereal brans; even then millet brans remain underutilized in research and industrial sections. Thus, promoting their use in development of low GI foods can assist in providing new healthy and nutrient dense alternatives.

### 2.2. Underutilized nuts/seeds

*Euryale ferox* commonly called as ‘Fox nut—also known as ‘Gorgon nut’, ‘Phool Makhana’, ‘Makhana’, ‘lotus seeds’, black diamond’ or ‘black gems’, are small edible black seeds obtained from foxnut fruit grown largely in India, and some south-east Asian countries like Nepal, Bangladesh, China, Malaysia, Philippines, Thailand, and Japan. In India major cultivation is taken place in Bihar (especially north east of Bihar) while other states like Manipur, Assam, Orissa, Kashmir, West Bengal also cultivate fox nuts (110–112). Traditionally, in ayurveda, these seeds are known to have medicinal properties and are effective against *Pitta* (bile disorders) and *Vata* (rheumatic disorders), it is also known for medicinal benefits in Unani (110, 111). These underutilized seeds are further processed to make snacks like popcorn. They are powerpack nutrient dense seeds having nutritional profile with higher amounts of minerals, amino acids (lysine+ arginine/proline ratio of 4.74–7.6%; amino acid index: 89–93%), while low saturated fats, calories, and glycemic index making them good snack source for population suffering from diabetes and cardiovascular disorders (110, 111, 113). Yang et al. (114) indicated that the fox nut starch had 10.13% RS content. Studies have also been conducted to increase the dietary fiber, RS and reduce total soluble sugars in makhana by modifying makhana starch enzymatically using amylopullulanase. The authors recorded increase in TDF (from 0.80 to 1.4%) and decrease in total soluble sugars (from 62.55 to 50.13 % mg) after enzymatic treatment (62). Thus, foxnut can be used as low GI snack and its flour can also be used in developing other value-added products.

### 2.3. Underutilized fruits, vegetable and their processing by-products

Fruits and vegetable by-products are generated at every stage of processing at industrial as well as household level. For instance, stalks of green leafy vegetables, either pomace, skin/peel or seeds or both from different vegetables and fruits like tomato, moringa, onion, mango, orange, jamun, apple, papaya, pomegranate, banana, lotus and so on that are discarded during processing. Just like the fruits and vegetables their by-products are a rich source of micro nutrients including vitamins, minerals, TDF and starch. It is suggested that unripe banana flour contains complex carbohydrates especially RS (up to 68% *w*/*w*), phenolic compounds, phytosterols and β-carotene thereby is a novel and functional ingredient in prevention of NCDs (115, 116). Raw/unripe banana (*Musa paradisiaca* L.) flours have been reported to have 66.5% RS, while 5.9% of slowly digestible starch (SDS) and 2.5% rapidly digesting starch thereby suggesting their application in developing low GI foods (117). High moisture treatments and storage have shown to improve the slow digestible starch and RS in unripe banana flour (118). Thus, unripe banana flours containing RS and indigestible CHO, are an important alternative ingredient to reduce GI of foods.

Research studies have also depicted evidence of using mango (*Mangifera indica*) seed/kernel flours given their fiber, starch and phytochemical profile (24). It was reported that mango kernel flour was good source of macronutrients like protein (7.53 g), crude fiber (2.20 g), fat (11.45 g), carbohydrate (69.77 g) and minerals like iron (12.4 mg), calcium (170 mg), zinc (5.60 mg), sodium (2.90 mg), potassium (368 mg), magnesium (210 mg), and copper (8.60 mg) (24). Similarly, seeds from apple (*Malus domestica*)—a major waste discarded during processing, are good in macro- and micro-nutrients (protein: 33.79–49.55 g/100 g; fat: 12–34%; ash: 3.66–5.20%; CHO: 23.50–29.80 g/100 g; calcium: 27–210 mg/100 g; iron: 11.0–27.1 mg/100 g; zinc: 4.4 mg/100 g; magnesium: 51–510 mg/100 g; potassium: 65–650 mg/100 g; sodium: 214.1 mg/100 g; phosphorous: 666.5 mg/100 g). Further, apple seed also had good lipid, amino acid and bioactive compounds like TPC (14.56–15.92 mg/g), TFC (154.16 mg RE/g) with phloridzin as a major bioactive bestowing therapeutic properties viz. anti-obesity, cardioprotective, hypoglycemic and antimicrobial properties. Thus, the researchers proposed apple seeds use in therapeutic and functional foods (20, 82–85).

Other agro-wastes i.e., peel of *Opuntia ficus indica* commonly called as prickly pear in from of flour can be used in developing nutrient rich biscuits as prickly pear peel flour (PPF) has good nutritional profile, containing 14.57% ash indicating presence of more minerals, generous quantities of fiber (20.70 g/100 g), 49.6 g/100 g of total CHO, higher content of polyphenols (2,776 mg/100 g), 3.3 g/100 g protein and 2.7 g/100 g crude fat. The PPF also had total carotenoids (10.90 mg βcarotene equivalent (CAR)/100 g), betacyanins (336.8 mg/100 g) and betaxantins (250.0 mg/100 g). The presence of bioactive pigments and compounds in PPF contributed to its radical scavenging activity (274.7 mmol/g eq. Trolox) (88). El-Beltagi et al. (90) also reported that PPF can be used as nutraceutical flour considering its rich bioactive (phenolic, flavonoid content) and nutritional profile with generous amounts of fiber, minerals like calcium, iron and zinc. They also reported that the PPF contained phenolic compounds like piscidic acid (8.89%), Feruloyl-D-glucose (10.01%), kaempferol (14.07%), 3-O-Methylquercetin (13.7%), isorhamnetin (27.1%) and eucomic acid (19.6%).

### 2.4. Dietary fiber and resistant starch

Dietary fiber(DF; viz. cellulose, hemicellulose, pectin, gum, lignin and others) is resistant to enzymatic digestion in the intestine, but fermentable in the colonic region. It is a phytochemical compound majorly concentrated in whole grains, cereal/millet brans, fruits and vegetables (11, 16, 119). Whole grain and DF consumption are linked to reduced risk of metabolic disorders like obesity, diabetes, cardiovascular diseases and even colonic cancers; however, DF consumption is still less than the recommended allowances (7). In India the recommended dietary allowances (RDA) for DF is 40 g/2,000 Kcal (91) yet the consumption is way below the RDA. In the current situation given the increasing burden of NCDs, consumers are choosing healthy food alternatives, thus researchers and food industrialists are taking efforts to develop healthy, nutrient and DF rich snacking alternatives. For instance, cereal bran like rice, wheat, oat, barley having dietary fiber are added in bakery products like biscuit, buns, muffins, cakes bread, and so on (15, 65, 107, 120, 121). Further, resistant starch (RS)—also considered as a type of DF, resists starch digestion that takes place by α-amylase and pullanase in the intestine and ferments in the colonic area (122).

Resistant starch (RS) viz. RS1 (e.g., present in cell wall of whole grains), RS2 (e.g., unripe banana, potatoes, Hylon^®^VII), RS3 (e.g., retrograded starch, Novelose^®^330), RS4 (chemically modified starch), and RS5 (e.g., starch with complex helical structures of lipids and amylose) are recently gaining research interest considering their health benefits in preventing and managing obesity, diabetes, and hyperlipidemia; and maintaining good colon health by developing short chain fatty acids, thereby maintaining beneficial colonic microbiota. Thus, they have been used as functional ingredient in developing low GI alternatives for various food products like bakery items; and also find application in developing low fat fat-replacers, emulsifying and thickening agents (123–127). There is abundant research being conducted on developing high RS plant varieties by implementing breeding activities (128). Even different food processing techniques are applied like autoclaving, repeated cooking and cooling to increase the resistant starch content in foods. It was reported by Zheng et al. (129) that underutilized proso millets grains when treated with hydrothermal and autoclaving treatment, resulted in increase of RS content to 12–15% as compared to the untreated grains. Researchers are engaged in developing novel food processing techniques and treatments to enhance the RS content of cereal and millet grains (129). For developing low GI and no gluten foods, even commercially available RS brands (viz., Fibersym^®^, Actistar^®^) are employed (130). A study reported use of RS4 (commercially available cross-linked starch) having 91.9% TDF and 83.3% RS to prepare low GI nutrient bar. Thirty-four grams of RS4 was added in wheat germ bar making its TDF (20 g/8) higher when compared to control wheat germ bar made using puffed wheat (TDF: 5 g) (131). Thus, inclusion of DF and RS in recommended quantities can be a preventive measure to control NCDs like obesity, diabetes, cardiovascular disorders; also given the production of short chain fatty acids they can be use prebiotic agents and also help in preventing risk of some cancers like colonic cancer (16, 70, 119).

Overall, it can be concluded that DF and RS have therapeutic benefits and can be used as functional ingredients in formulating low GI therapeutic food products.

## 3. Application of novel ingredients and starch in low glycemic index (GI) food formulations

**Figure 1:**
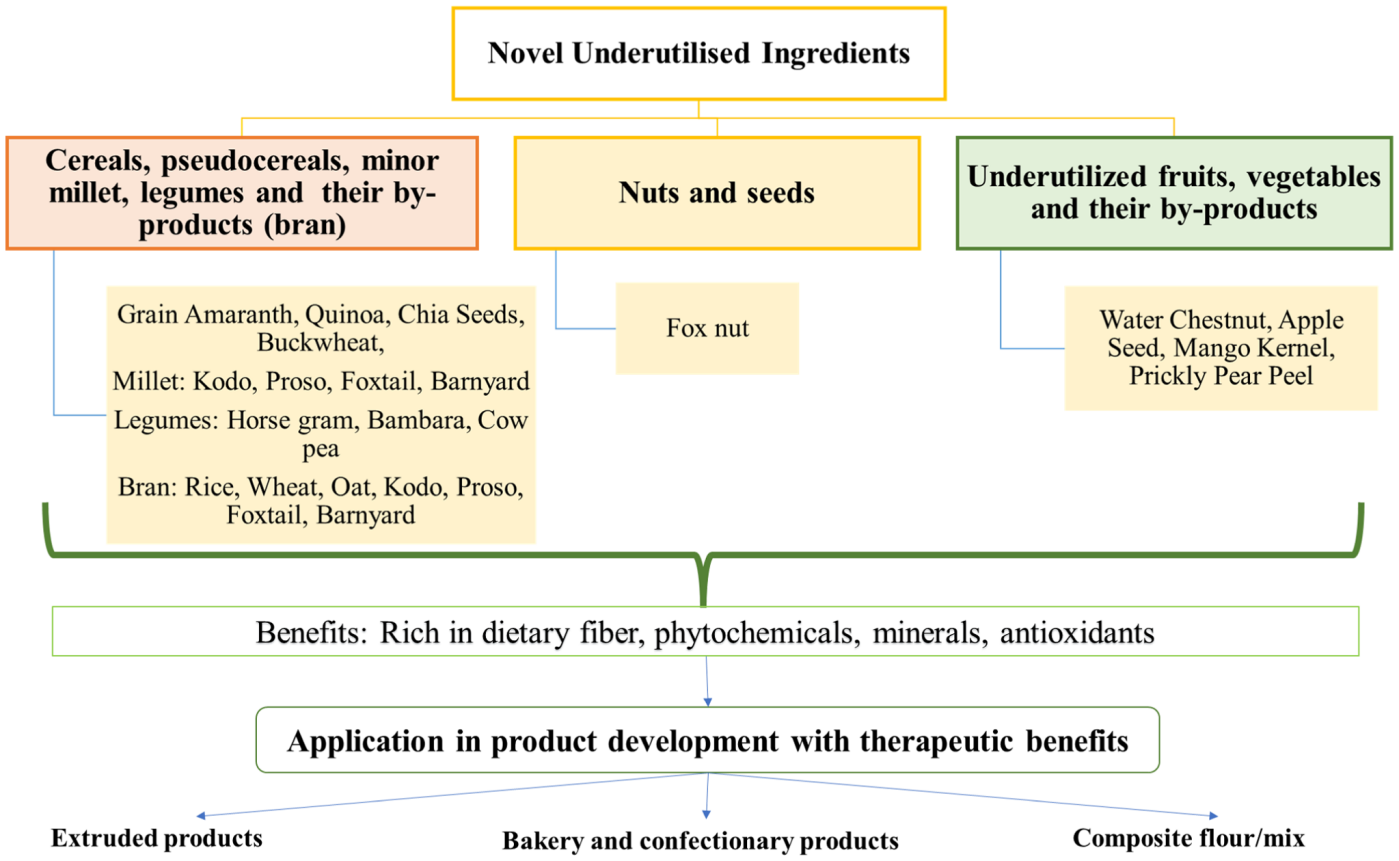
Application of novel ingredients in therapeutic product development.

This section compiles scientific literature on developing low GI foods namely: extruded snacks, bakery and confectionary and composite mixes, with therapeutic benefits by supplementing the different above-mentioned novel ingredients. It also delineates the effects of value addition on their sensory, physical, nutritional and functional properties. Glycemic index of selected novel ingredients, their by-products and their products have been listed in Table 2. Figure 1 summarizes the application of novel ingredients in food formulations.

**Table 2:** Glycaemic index of novel food sources and some of their selected products.

Novel food sources	Glycaemic index	References
Pseudocereals		
Amaranth	Grain: 87, 47.65 eGI Puffed grain: 101 Flour (coarse): 97 Flour: 66.12 (predicted GI) Extruded Breakfast cereal (EBC): Unmalted Amaranth flour EBC: 90, Malted Amaranth flour EBC: 85 Extruded snack (amaranth+soy+shallot): 34.37–38.75 Bread (amaranth+sweet potato): 51.8 Multigrain snack bars (amaranth+acha+pearl millet): 48.63–57.11	Urubkov et al. (132); Ojedokun et al. (133); Di Cairano et al. (98); Arslan-Tontul et al. (100); Olagunju et al. (96); Olagunju et al. (134); Calderón de la Barca et al. (39)
Quinoa	Grain: 35–53, 61.50 eGI Flour: 77.06 (predicted GI)	Bastidas et al. (135); Lutz and Bascuñán (136); Di Cairano et al. (98); Arslan-Tontul et al. (100)
Chia	Chia seeds: 28.53 eGI Bread prepared with different concentration (5, 10%) of chia seeds: Chia seeds: 91.6, 80.5 Chia whole flour: 86.1, 75.0 Semi-defatted chia flour: 88.4, 77 Low-fat chia flour: 87.3, 76.3	Miranda-Ramos et al. (137); Arslan-Tontul et al. (100)
Buckwheat	Grain: 52.35 eGI Flour: 50.74 (predicted GI) Biscuit (buckwheat (50%), millet (50, 30%), sorghum (50, 30%) and chick pea (20%) or lentil (20%)): 55.07–63.18	Di Cairano et al. (98); Di Cairano et al. (138); Arslan-Tontul et al. (100)
Millet		
Kodo millet	Flour: 32.47 eGI Grain 65.4	Annor et al. (139); Anitha et al. (140)
Proso millet	100% proso millet products: 50–65 (eGI): couscous: 50.2, porridge: 53.1, Muffin: 56.0, extruded snack: 64.7. Whole grain millet couscous: 28.5	Mcsweeney (141); McSweeney et al. (142); Das et al. (45)
Foxtail millet	Grain: 54.5 Cooked millet: 64.4 Foxtail millet porridge: 93.6 Steamed bread: 89.6 Millet pancake: 83–76.2 Millet cookies (15% foxtail millet, 15% arrowroot flour, and 30% of kidney beans flour): 37.6 Foxtail millet *dosa*: 59.25	Ren et al. (143); Narayanan et al. (144); Lestari et al. (145); Anitha et al. (140)
Barnyard millet	Dehulled grain: 45.2 to 54.8 Dehulled and heated grain: 38.4 to 45.3 Grain: 42.3	Ugare et al. (146); Anitha et al. (140)
Legumes		
Horse gram	39.83-39.64 (predicted GI)	Eashwarage et al. (147)
Bambara beans	Bambara bean: 40.1 Pudding: 40.13	Nnadi and Keshinro (148); Singh et al. (149); Oyeyinka et al. (150)
Cow pea	41.34–42.23 (predicted GI) Boiled cowpea paste: 54–56 Boiled cowpea: 6–11 Boiled and cooked (brown, white and black): 29–46.64 Grain (white, brown and black): 29–41	Oboh and Agu (151); Oboh et al. (152); Akinlua, et al. (153); Eashwarage et al. (147); Singh et al. (149)
Nuts and seeds		
Fox nut	Roasted: 37.05	Liaquat et al. (63)
Cereal and millet by-products		
Rice bran	25% rice bran incorporated wheat chapati: 52.40 25% rice bran incorporated wheat rava dosa: 46.60 25% rice bran incorporated wheat dosa: 52.81 Bread developed using rice bran soymilk (RBS): 83.1 Rice bran arepa (bread): 85.96	Schnell et al. (154); Premakumari et al. (155); Camps et al. (156);
Wheat bran	Wheat bran (low, moderate, high protein): 25–21 Wheat bran: 45–50 Autoclaved wheat bran: 105–118 Wheat bran pasta: 50	Rico et al. (157); Naji-Tabasi et al. (158); Jimenez-Pulido et al. (159)
Kodo bran	30% kodo bran muffin: 56.42	Barbhai et al. (14)
Foxtail bran	20% Foxtail bran bun: 57.71	Barbhai et al. (14)
Underutilized fruits, vegetables and their by-products		
Water chestnut	Crackers (70% waterchestnut + 30% barley): 30.16	Hussain et al. (81)
Unripe banana	Cookies substituting unripe banana flour (UBF) at 15, 30, 50 percent: UBF15: 115.2 UBF30: 106.5 UBF50: 98.6	Agama-Acevedo et al. (160)
Mango seed/kernel	Mango seed starch: 48.8–50.9	Sandhu and Lim (161)

### 3.1. Extruded products

Research indicates that underutilized cereals are used in development of extruded flours and find utilization as novel flour for development of various extruded or baked products (162). Ojedokun et al. (133) used blends of malted amaranth and roasted sesame for developing extruded breakfast cereal and stated that formulation with 50% amaranth flour and 50% sesame were best acceptable. This formulation of breakfast cereal had the highest TDF (6.23 g/100 g) which reduced the GI and glycemic load (GL) of the product. Even the amino acid profile was better with addition of grain amaranth and sesame than control breakfast cereal (133). Optimized quantities of amaranth (55%), shallot (25%) and soycake (20%) were combined for preparing nutrient rich extruded snack; and their sensory acceptability was above 7. It was observed that the different variations of extruded snacks developed using amaranth, shallot and soycake had higher magnesium (42.00–46.10 mg/100 g) than wheat shallot market sample used as control (28.60 mg/100 g) and lower GI ranging between 34.37 and 38.75. Thus, the scientists indicated that these products find best suitability as alternative snacks for hyperglycemic patients (134).

Cassava-vegetable pasta using leafy vegetables namely amaranth leaves and fluted pumpkin leaf (at conc. 5, 10%) was prepared by Lawal et al. (163). It was observed that nutritional content improved with the addition of leafy vegetables than control. The protein content nearly doubled from 0.99 g/100 g (control) to 2.95 g/100 g (10% fluted pumpkin leaf-fortified cassava pasta), and dietary fiber increased from 9 g/100 g (control) to 10 g/100 g (10% fluted pumpkin leaf-fortified cassava pasta). The TPC, TFC and β-carotene ranged between 445–1,098 μg GAE/g; 58–61.6 μg RE/g and 2.11–7.82 μg/g in vegetable fortified pasta while control had only 226.6 μg GAE/g; 34.1 μg RE/g; and 0.48 μg/g, respectively. The resistant starch increased from 1.12% in control to 1.78 2.45% in vegetable fortified pasta thereby decreasing its GI from 71.72 (control) to 58.14–61.39 (vegetable fortified pasta). Thus, it was evident that underutilized leafy vegetable incorporation can reduce the GI of pasta and increase its nutritional value.

### 3.2. Bakery and confectionary products

Cereals, fruits and vegetable and their byproducts are used for value addition of baked products like biscuits, cookies, cakes and others (16, 25, 88, 164, 165). Underutilized roots and tubers like water chest nut serves as functional ingredient in reducing GI of crackers. Low GI crackers using combination of water chestnut (*Eleocharis dulcis*) and barley flour (100:0, 70:30, 50:50, 30:70, and 0:100, respectively) were developed by Hussain et al. (81). They concluded that 70% water chestnut flour and 30% barley flour (70, 30) was best accepted with overall acceptability of 4.55 on 5-point hedonic scale and had low GI of 30.16 and 13.47 glycemic load. Crackers developed with 70:30 (water chestnut: barley flour) combination had crude fiber content of 3.80%, 51.9% carbohydrate, 7.21% dietary fiber and 2.30% β-glucan; this could have reduced the GI of the crackers (81). Similarly, Agama-Acevedo et al. (160) lowered the glycemic index of cookies by addition UBF: unripe banana flour (at 15 g, 30 g, 50 g per 100 g), because UBF addition increased the resistant starch content of cookies to 3.1 g/100 g (UBF: 15 g/100 g), 5.44 g/100 g (UBF: 30 g/100 g) and 8.37 g/100 g (UBF: 50 g/100 g) while control cookies only had 2.3 g/100 g. The DF and SDS also enhanced in cookies with UBF (TDF: 6.6–10.9 g/100 g; SDS: 8.7–10.9 g/100 g) than control (DF: 4.8 g/100 g; SDS: 8.3 g/100 g) cookies. This lowered the predicted GI of cookies with UBF (UBF15: 115.2, UBF30: 106.5 and UBF50: 98.6) than control cookies (116.6).

Miranda-Ramos and Haros (37) optimized bread formulation by substituting combination flour containing underutilized pseudocereals i.e., amaranth (20%), chia (10%) and quinoa (4%) in wheat flour. Replacement of wheat flour with combination flour increased the protein (control: 14.3% optimized bread: 15.8%), and TDF (control: 5.4%; optimized bread: 15%) content. The increase TDF content resulted in decreased energy values of optimized bread when compared to control bread and it also decreased the loaf-specific volume. Addition of these pseudocereal made the optimized bread darker; however, the overall acceptability was similar for whole bread (8.1) and optimized bread (8.7). Calderón de la Barca et al. (39) also designed value added bread by substituting amaranth (22.7%) and sweet potato (8.6%) flours in wheat (68.7%) having sensory acceptability on par with control (wheat bread). This substitution of amaranth and sweet potato significantly enhanced nutritional and bioactive profile of bread compared to control bread with protein (10.15%), ash (2.44%), lipids (3.0%), dietary fiber (4.98 g/100 g), TPC (83.13mg GAE/100 g), DPPH (67.5%) higher than control bread. Especially, the beta-carotene content improved significantly from some traces in control bread to 1123.2 μg/100 g in value-added bread. The authors also confirmed reduction in GI of bread upon value addition with amaranth and sweet potato (51.8) when compared to control bread (GI: 72.0). In a study (96), multigrain snack bar (SB) was developed by addition of amaranth (A), acha (*Digitaria exilis*; DE) and pearl millet (PM) at different concentrations (viz. SB1 A90%:DE5%:PM 5% and SB2 A47.98%: DE26.68%: PM25.34%) and oat bar was used as control. All the snack bars were liked by the semi trained panel member, on 9-point hedonic scale with overall acceptability above 6–7 i.e., 6.75 (SB1), 7.15 (SB2) and 8.75 (control). The multigrain snack bar had higher nutritional profile for protein, ash, iron and zinc when compared to control; while calcium was higher in SB1 than control, but SB2 had lower calcium levels. Presence of phytate in large quantities can hinder the absorption of minerals, especially for calcium, iron, zinc and potassium due to binding of phytate with minerals. Usually, the phytate is not degraded easily, however, its levels can be reduced by using different processing techniques like soaking, roasting, milling, extrusion, fermentation, or use of phytase enzymes (166, 167). Despite the higher phytate and oxalates in SB1, and SB2 than control snack bar, the molar ratios for phytic acid with zinc, calcium, iron, potassium were well within the acceptable ratios for the multigrain snack bars (SB1 and SB2). The DPPH scavenging activity, ABTS activity, ferric reducing power, metal chelating ability were higher in SB1 and SB2 than control. Along with these nutritional benefits SB1 (48.63) and SB2 (57.11) also had lower GI than control (69.81) (96). Another study also indicated that combination of 15% foxtail millet, 15% arrowroot flour, and 30% of kidney beans flour for preparing cookies served best in terms of sensory acceptability and had low GI (37.6) owing to its high DF (14.48%) and RS (9.67%) content (145).

Cookies were developed using 4-6% grape pomace having higher total phenol of 3.42–4.03 mg GAE/g, protein (5.25–5.69%) and fiber (2.04-2.13%) than control cookies (TPC: 3.14 mg GAE/g; Protein: 3.10%; fiber: 1.74%). The cookies had darker color upon addition of grape pomace resulting in decreasing sensory scores of panel member; however up to 4–6% the grape pomace was acceptable (165). Similarly, de Toledo et al. (164) developed cookies using byproducts of melon (i.e., peels), pineapple (i.e., central axis) and apple (endocarp) at 5, 10, 15% concentrations. The nutritional composition became better as the concentration of the fruit byproducts increased. The cookies made with 15 % melon, pineapple and apple flours had higher fiber (soluble: 2.92, 1.05, 1.64% and insoluble: 3.54, 2.08, 2.52, respectively) than control (1.20%; 1.45% respectively). The protein content also increased from 8.54% (control) to 8.94% (15% melon); while carbohydrates (%) decreased from 19.55 (control) to 17.88 (15%pineapple); 19.06 (15% apple) and 18.81 (15% melon). Considering the sensory scores, 15% pineapple byproduct enriched cookies were highly acceptable, followed by 15% apple- and 15% melon-by products. Another nutritionally rich biscuits developed using prickly pear peel flour (20–30 g/100) scored higher overall sensory scores even when the color of biscuits darkened compared to control. The functional properties of flour like water absorption and holding capacities increased with increasing addition of prickly pear peel flour (PPF), due to their higher fiber content. However, the rheological properties of dough indicated that hardness increased with addition of PPF. Nutritional analysis of PPF biscuits (PPF: 30 g/100 g) showed improvement in ash (3.62 g/100 g), fiber (3.14 g/100 g), TPC (575 mg GAE/100 g), radical scavenging activity (236 mmol/g eq. Trolox), total carotenoid (3.54 mg CAR/100 g) and total betalains (176.7 mg/100 g), while decreased CHO (62.5 g/100 g), fat (15.3 g/100 g) and energy (1,726 Kcal) compared to control (ash:0.86 g; fiber: 0.77 g; TPC: 34 mg GAE/100 g; radical scavenging activity: 141 mmol/g eq. Trolox; total carotenoids: 0.90 mg CAR/100 g; total betalains: not detected; CHO: 63.5 g; fat: 16.4 g; energy:1,766 Kcal/100 g) respectively (88). Likewise, El-Beltagi et al. (90) also developed cakes with 5–15% PPF replacement in wheat flour and found that the fiber, mineral, phenols, and flavonoid content increased in cake resulting in higher antioxidant activity in the cake. They concluded that sensory scores were higher for 10% PPF enriched cake.

Research indicated use of commercial and laboratory-based RS in product development for reducing the GI or preparing low GI bakery products. Kahraman et al. (126) developed low GI cookies, by addition of different sources like wheat bran, lab-scale produced cross-linked starch (viz., cross-linked wheat starch: XL-W; cross-linked corn starch: XL-C), and commercial RS sources (viz., Hylon VII, Novelose330, Fibersym) at different concentration levels (0, 25, 50, and 75%). They indicated that addition of wheat bran, XL-C, Hylon VII, Novelose330, and Fibersym decreased the spread ratio and increased the thickness of the cookies when compared to control cookies (without any supplementation). In terms of TDF with increase in the supplementation levels of XL-W, XL-C, Fibersym and wheat bran, the TDF increased from 1.0 to 32.4%; 1.0 to 18.0%; 1.0 to 25.1%, and 1.0 to 27.2% respectively. This increase in TDF also in turn reduced the *in vitro* GI of the developed cookies (XL-C:78.8; XL-W: 77.2; Hylon VII: 83.0; Novelose 330: 86.2; Fibersym: 81.7; Wheat bran: 88.1) upon comparison with control (112.1). Rakmai et al. (1) developed low GI (49.3-51.9) pan cakes by partially replacing rice flour (Jasmine or Sangyod) with resistant maltodextrin (RM) at 10, 20 and 30% concentration and sucrose with sucralose (50 and 25%). They found that addition of RM decreased the chewiness and gumminess of pancakes. RM added pancakes were softer, however their firmness decreased as the concentration of RM increased above 20%. Thus, at level one they found that 10% RM replacement was most acceptable. At level two, the researchers partially replaced the sucrose used in making pancake with low calorie sugars –sucralose or stevioside. Their findings revealed that pan cakes (either made from Jasmine rice or Sangyod rice) with 25% sucralose replacing sucrose scored more (Jasmine + sucrose: 5.6; Sangyod + sucrose: 3.4) compared to stevioside pancakes (Jasmine + stevioside: 4.4; Sangyod + stevioside: 3.1) on 9-point hedonic scale. Further, the sensory score for overall acceptability was higher for Jasmine+10%RM+25%sucrose (6.7) than Sangyod + 10%RM + 25%sucrose (4.6) and control (Jasmine control: 5.7; Sangyod control: 3.5).

### 3.3. Composite flour/mix

Composite flour developed with addition of 20% wheat bran, enriched iron (29.2%), phytic acid (1.09 g/100 g), protein (12.49%), fat (2.45%), fiber (2.51%) and ash content (1.89%) indicating that nutritional profile enhanced with addition of bran (69). Naseer et al. (168) optimized instant *phirni* mix using 70% skimmed milk powder (SMP), 30% high amylose rice (HAR) and 0.8% CMC (carboxymethyl cellulose) and their overall sensory score was 8.39 on 9-point hedonic scale. The physio-chemical and nutritional profile of optimized *phirni* mix (per 100 g) had higher protein (25.12 g), ash (7.12 g), dietary fiber (3.10 g), and amylose (15.31 g) content than control (market *phirni* mix) sample (9.56 g; 6.08 g; 0.67 g; 5.27 g respectively). The addition of HAR, CMC also decreased the carbohydrate (60.58 g), sugars (30.00g), fat (1,30g), and energy (354.50 Kcal) than the control sample (77.91 g; 50.25 g; 2.14 g; 369.14 Kcal respectively). Upon reconstitution of optimized *phirni* mix, RS (4.38 g), hydrolysis index (15.31), predicted glycemic index (48.12) and glycemic load (7.50) were found decreased in the optimized mix than reconstituted control *phirni* mix (0.50 g; 37.32; 60.20; 9.78 respectively). Further, Di Cairano et al. (138) developed six variations of composite biscuit flour by combining buckwheat (50%), millet (50%, 30%), sorghum (50%, 30%) and chick pea (20%) or lentil (20%). The biscuits prepared from these composite flours were having RS content ranging between 0.30–0.73 g/100 g which was higher to control (0.40 g/100 g) and total starch ranging between 34.23–38.63 g/100 g which was lower to control (50.99 g/100 g). even the predicted glycemic index decreased in composite flours from 70.97 (control) to 55.07–63.18.

The above findings revealed that use of novel ingredients and unconventional food sources at different concentrations can be implemented to reduce GI of extruded, bakery products, and composite flours/mixes without affecting their sensory characteristics.

## 4. Therapeutic properties of products developed using novel ingredients and starch

This section provides brief insight on the therapeutic benefits of low GI products developed using the above mentioned selected novel ingredients and/or resistant starch. These low GI products can be considered as therapeutic alternative snacks for population suffering from NCDs given their hypoglycemic, hypocholesterolemic and anti-obesity properties.

### 4.1. Anti-obesity and hypolipidemic properties

Fu et al. (169) indicated that 6-week supplementation of banana resistant starch (BRS) at low (1.25 g/kg), medium (2.50 g/kg) and high (5.0 g/kg) dose along with high fat diet (HFD), fed to obese rats could prevent rise of glucose (4.16–3.78 mmol/L) when compared to control (fed with HFD only) obese rats (9.77 mmol/L). Even the triglyceride, total cholesterol, and low-density lipoprotein levels were lower in BRS + HFD obese rats (0.44–0.47 mmol/L; 1.45–1.61 mmol/L; 0.49–0.33 mmol/L respectively) than control obese rats (0.65; 1.79; 0.48 respectively). The authors also indicated that the BRS improved the gut microbiota by increasing the ratio of *Bacteroidetes/Firmicutes* microorganism. Even the serum level of leptin and insulin decrease in rats fed with BRS (leptin: 1.82–1.37 ng/ml; insulin: 11.51–9.29 U/L) than the control rats (leptin: 2.10 ng/ml; insulin: 15.15 U/L). The ghrelin hormone level which was less in obese rat (0.76 mU/L) was improved in rats fed with BRS (0.79–0.92 mU/L). Adiponectin a hormone related to anti-diabetic and cardioprotective activity was low in obese rats (23.60 ng/ml) which was increased to 24.71–34.44 ng/ml in BRS fed rats. Thus, it was concluded that BRS demonstrated anti-obesity effect by regulating the glucose and lipid mechanism, reducing the serum hormonal levels of leptin and insulin and increasing the ratio of beneficial gut microbiota (169).

The hypolipidemic properties of consuming RS4 enriched flours was delineated by Nichenametla et al. (170). They indicated that upon 12 weeks consumption of control (RS: 2 g/100 g) and RF4 enriched flours (RS: 25 g/100 g), RS4 enriched composite flour decreased total cholesterol by 7.2%, and the low-density lipoprotein by 5.5% in human subjects (male and female) with metabolic syndrome. Thus, the authors suggested that adding RS4 to daily regular diets can improve dyslipidemia and prevent the risk of metabolic syndrome and associated cardiovascular disorders (CVDs). This encourages the use of these novel ingredients and starches for harnessing therapeutic benefits.

### 4.2. Hypoglycemic properties

Low GI and GL foods are known to have hypoglycemic effect, thus are recommended for diabetic patients as part of their diets. Management of diet plays a major role in preventing and maintaining normal blood sugar levels of a diabetic person (171). The current trend of snacking includes consumption of highly processed, high calorie foods, mainly bakery products, fried foods that lack fiber or other essential nutrients and also fall into high GI category. However, efforts are being taken to reduce the GI of some of these snacks like bakery products, by value adding or enriching these products with nutrient dense novel ingredients that are rich source of fiber and bioactive compounds apart from normal nutrients. When white bread enriched with oat fiber (insoluble fiber 10.4 g per portion) was supplemented to overweight and obese women for 3 days, their insulin sensitivity improved. Serum insulin was noted as 29.7 in experimental group while it was 32.3 pmol/l in control (172). Miranda-Ramos and Haros (37) developed bread by addition of amaranth (20%), chia (10%) and quinoa (4%) in wheat flour; which reduced the GI of optimized bread (85%) upon comparison with control (95%). This reduction in GI can be correlated to the GI of quinoa, amaranth and chia in the formulation due to high dietary fiber and decreased starch content. Further, addition of pseudocereals also reduced the starch hydrolysis at 90 mins in optimized bread (68.1%) as compared to control bread (84.6%) thereby providing hypoglycemic effect.

Research indicates development of low GI foods by adding fruits and vegetable by-products. The fruits by products like mango peel were implemented for reducing GI of foods as they are rich in dietary fiber and phenols. Ajila et al. (173) revealed that value addition of mango peel (5–20%) in biscuits increased the TDF content to 20.7% while control only had 6.5%. Similarly, the polyphenol content was higher in 20% mango peel incorporated biscuits (4,500 μg GAE/g) than control (540 μg GAE/g) thereby, making it richer in antioxidant and bioactive compounds. Silva et al. (174) indicated that unripe banana pasta (75%) when fed to diabetic rats was able to prevent the hyperglycemia and also decreased cholesterol and triglyceride levels compared to the control diabetic group.

Addition of dietary fiber or RS has also proven to lower the GI of foods and provide hypoglycemic effect. A study has depicted that supplementation of nutri bar containing 34 g RS4, lowered the glucose and insulin response as compared to bar made with puffed wheat till 120 minutes after consumption (131). Rakmai et al. (1) indicated that pan cakes made by substituting rice flour (Jasmine or Sangyod) with 10% resistant maltodextrin (RM) and sucrose with 25% sucralose decreased the GI of pancakes to 51.9 (Jasmine + 10%RM + 25%sucrolase) and 49.3 (Sangyod+ 10%RM+ 25%sucrolase) when compared to control (Sangyod control: 58.2; Jasmine control: 60.8). The authors concluded that addition of dietary fiber and low-calorie sweetener reduced the calorific value (305.59 Kcal), carbohydrate (39.16g) and increased the dietary fiber (0.93 g), protein (6.39 g) content, compared to control pancakes (Protein: 5.75 g; TDF: 0 g; CHO: 46.59 g) thus making it an alternative for high GI pancakes.

Similarly, a promising low GI alternative for available milk desserts (*phirni*) for diabetic population was developed by Naseer et al. (168) combining skimmed milk powder (70%), high amylose rice (30%) and 0.8% carboxymethyl cellulose. Further, a study conducted by Kahraman et al. (126) also confirmed that addition of crosslinked (XL) corn (C) or wheat (W) starch and wheat bran reduced the GI of cookies (XL-C;78.8; XL-W: 77.2; wheat bran:88.1) than control (112.1) and thus, depicting the hypoglycemic properties of bran and crosslinked starch. Altogether, it is evident from these research studies that novel ingredients have complex carbohydrates and starch components thus, can be harnessed in developing low GI foods serving as alternatives for regular commercially available snacks consumed by the population suffering with NCDs.

## 5. Conclusion and future scope

With the increasing prevalence of NCDs across the globe, coupled with a sedentary life-style, it has become a pressing priority to develop low GI alternatives for regular snacking foods. This review brought to the foreground a few selected underutilized novel ingredients and unconventional starches that can be harnessed for lowering the GI of different bakery, extruded products or even composite mixes. Exploring different unconventional food sources and starch relates to a sustainable way of using underexploited grains, fruits, vegetables and agro-wastes/by-products. The review highlighted nutritional profile of such pseudocereals, millets and their by-products, fruits and vegetable by-products, delineating that they are rich sources of dietary fiber, complex slow digesting starches, RS and phytochemicals; thereby can aid in reducing/lowering the GI of foods. These novel foods can also provide therapeutic benefits like antidiabetic, anti-obesity, and hypoglycemic effects. Further, there is a need to commercialize and create an extensive market for popularizing such therapeutic low GI foods at commercial industrial level. Ample evidence is available on product development however, additional research on clinical trials is required for analyzing the complete nutritional and nutraceutical effect of these major ingredients upon consumption and assessing their glucose and/or lipid lowering mechanisms for gaining definitive results.

## Author contributions

All authors listed have made a substantial, direct, and intellectual contribution to the work and approved it for publication.

## Conflict of interest

The authors declare that the research was conducted in the absence of any commercial or financial relationships that could be construed as a potential conflict of interest.

## Publisher’s note

All claims expressed in this article are solely those of the authors and do not necessarily represent those of their affiliated organizations, or those of the publisher, the editors and the reviewers. Any product that may be evaluated in this article, or claim that may be made by its manufacturer, is not guaranteed or endorsed by the publisher.
